# Enhancement of Magnetoelectric Effect in Layered Polymer Composites by Zn^2+^ and Ni^2+^ Substitution in CoFe_2_O_4_ Nanoparticles

**DOI:** 10.3390/polym17091183

**Published:** 2025-04-26

**Authors:** Liudmila A. Makarova, Michail T. Musaev, Margarita R. Kalandiia, Sergey A. Kostrov, Elena Yu. Kramarenko, Vitalii D. Salnikov, Damir E. Gavrilov, Aleksander S. Omelyanchik, Valeria V. Rodionova, Nikolai S. Perov

**Affiliations:** 1 Faculty of Physics, Lomonosov Moscow State University, 119991 Moscow, Russia; musaev.mt21@physics.msu.ru (M.T.M.); mr.kalandiya@physics.msu.ru (M.R.K.); kram@polly.phys.msu.ru (E.Y.K.); perov@magn.ru (N.S.P.); 2 A.N. Nesmeyanov Institute of Organoelement Compounds of the Russian Academy of Sciences (INEOS), 119334 Moscow, Russia; sergeykostrov1996@gmail.com; 3 REC Smart Materials and Biomedical Applications, Immanuel Kant Baltic Federal University, 236041 Kaliningrad, Russia; vdmsalnikov2@kantiana.ru (V.D.S.); damixxxmag@gmail.com (D.E.G.); aleksander.omelianchik@ext.unige.it (A.S.O.); vvrodionova@kantiana.ru (V.V.R.)

**Keywords:** cobalt ferrite nanoparticles, magnetic elastomer, piezopolymer, magnetoelectric effect, polymer multiferroic

## Abstract

Two-layered structures consisting of piezopolymer and magnetic elastomer were investigated as magnetoelectric material. Three types of magnetic elastomer based on cobalt ferrite (CoFe_2_O_4_) and Ni- or Zn-substituted CoFe_2_O_4_ nanoparticles were used as magnetically sensitive layers. Cobalt ferrite nanoparticles are considered one of the most promising metal-oxide nanomaterials because of their favorable magnetic properties, such as high saturation magnetization and magnetic anisotropy. The substitution of Co^2+^ in cobalt ferrite with other transition metals allows for additional tailoring of these properties. The modified magnetic behavior of the substituted CoFe_2_O_4_ nanoparticles directly influenced the magnetic properties of magnetic elastomers and, consequently, the magnetoelectric response of composite structures. In this case, the resonant frequency of the magnetoelectric effect remained largely independent of the type of magnetic nanoparticles in the magnetic elastomer layer but its magnitude increased upon Zn substitution up to ~107 mV·cm^−1^·Oe^−1^. These findings highlight the potential of chemically engineered magnetic properties of CoFe_2_O_4_ nanoparticles for manufacturing magnetoelectric composites to expand their applications in energy harvesting and sensors.

## 1. Introduction

Multiferroics are materials that simultaneously exhibit at least two ferroic orders—namely, ferromagnetic, ferroelectric, ferroelastic—and demonstrate coupling between them [1]. In particular, magnetoelectrics have both ferromagnetic and ferroelectric ordering concurrently. The ability to convert energy between electric and magnetic fields classifies them as multifunctional materials, which has attracted considerable interest [1,2,3,4,5,6]. Research into multiferroics enhances the horizons of electronics, including microwave devices and sensors [7,8,9,10,11]. Furthermore, the applications of multiferroics are extensive and include multiple-state memories [12,13], data storage and switching [14], organic optoelectronics [15,16], spintronics [17,18], charge transfer-based sensitizers for photovoltaics [19], tunable microwave devices [10], energy harvesting [20], microelectromechanical systems (MEMS) [21,22], and others.

Single-phase multiferroics show magnetoelectric effects due to the presence of coupled ordered phases within the crystal lattice (10^−5^–10^−3^ V·cm^−1^·Oe^−1^) [23]. A significantly higher magnetoelectric effect (1–100 V·cm^−1^·Oe^−1^) can be achieved in composite multiferroics based on piezoelectric and magnetostrictive materials [23,24]. The direct magnetoelectric coefficient is defined as the ratio of the induced electric field intensity to the applied magnetic field intensity. In composite multiferroics, the mechanism of magnetoelectric conversion is based on the deformation of the magnetostrictive component under an external magnetic field, which in turn mechanically deforms the coupled piezoelectric component. Every practical application of multiferroics necessitates rigorous optimization of parameters such as intercomponent coupling, operating frequency, input signal amplitude, geometric dimensions, and sensitivity to external stimuli [25,26,27,28,29].

For instance, in nanocomposite multiferroics, cobalt ferrite nanoparticles are actively employed as the magnetostrictive phase, while bismuth ferrite or barium titanate nanoparticles serve as the ferroelectric component [29,30,31,32,33]. Cobalt ferrite and its modifications exhibit high chemical stability, strong magnetocrystalline anisotropy, and a significant magnetostrictive effect [32,34]. Depending on the composite geometry—ether in the form of fibers [32] or pressed tablets [33]—the magnitude of the effect and the potential application areas of these magnetoelectrics vary with the relative content of the two phases. By varying the sample shape, the types of piezoelectric and magnetostrictive materials, and the configurations of the external magnetic fields, the sensitivity of sensors can be modulated [6,11,29].

Another example of composite multiferroics is provided by layered materials. Layered multiferroics based on piezoceramics and magnetostrictive metals are actively investigated and exhibit high magnetoelectric coefficients and sensitivity [35,36,37,38]. However, employing a polymer with ferroelectric properties as the piezoelectric layer enables the development of devices for flexible electronics [39,40,41,42,43,44,45,46,47,48,49,50,51,52]. The piezopolymer polyvinylidene fluoride (PVDF) and its copolymer modifications are most commonly used in studies of the magnetoelectric effect [53]. A key aspect in the fabrication of such polymers is the formation of a stable β-phase in PVDF, which is responsible for its ferroelectric properties. It has been demonstrated that the addition of nanodispersed fillers creates additional nucleation centers during the polarization of PVDF and increases its piezoelectric coefficient [51,54]. Multiferroics based on PVDF filled with magnetic nanoparticles, such as cobalt ferrite, are of considerable interest [44,45,46,47,48,49]. Layered structures employing PVDF films include configurations with a pure PVDF layer combined with a metallic magnetostrictive layer as well as PVDF-based polymer layers with incorporated magnetostrictive and ferroelectric nanoparticles [39,40,41,42,43]. Such polymer composite multiferroics can be utilized for energy harvesting devices [21,55,56]. Devices capable of harvesting energy from environmental sources are of enormous commercial interest because they can power wireless sensors and sensor networks, environmental monitoring systems, structural health monitoring systems, security systems, medical implants, and portable electronics [57]. One of the intriguing outcomes is the development of self-biased composites, in which the magnetoelectric effect is observed even in the absence of an external constant magnetic field [51].

One of the proposed types of layered polymer multiferroics is the composite structure comprising a piezopolymer layer and a magnetic elastomer or magnetoactive elastomer (MAE) layer [58,59,60,61,62,63,64]. If iron microparticles are used as fillers in the magnetic elastomer, the magnetodeformational effect—analogous to the magnetostrictive effect in metals—leads to bending of the structure due to the differing stiffnesses of the layers and their integral mechanical coupling, thereby producing a magnetoelectric effect [61,63,64]. Moreover, for the first time, a magnetoelectric effect was achieved due to the mechanical instability of the magnetic elastomer layer in transverse magnetic fields, with record-high values of up to 160 V·cm^−1^·Oe^−1^ [64].

The ability to generate an electrical voltage in the absence of an external constant magnetic field has also been demonstrated for such multiferroics containing iron microparticles. Specifically, a gradient alternating magnetic field produced by a single electromagnet bends the structure due to the force acting on each particle in the magnetic elastomer layer (f~m·∇H) [60,62].

Among various magnetic materials, nanoparticles of spinel ferrites with the general formula Me^2+^Fe_2_O_4_ (where Me^2+^ is a divalent transition metal) are particularly attractive due to their chemical versatility, stability, and the ability to fine-tune their magnetic properties through cation substitution [65]. Cobalt ferrite (CoFe_2_O_4_) is of particular interest due to its excellent combination of high magnetocrystalline anisotropy and moderate saturation magnetization, making it a promising candidate for magnetoelectric composite applications [66,67]. High magnetocrystalline anisotropy is attributed to the strong single-ion anisotropy of Co^2+^ ions in octahedral sites arising from unquenched spin–orbit coupling. Substituting Co^2+^ with other metal ions allows the modulation of the magnetic properties of these ferrites. For instance, substitution with Ni^2+^, which also prefers the octahedral sites, typically results in reduced saturation magnetization and magnetic anisotropy [68]. In contrast, Zn^2+^ preferentially occupies the tetrahedral sites, pushing Fe^3+^ ions to the octahedral sites. At moderate concentrations (<50 at.%), this redistribution enhances the net magnetic moment by increasing sublattice imbalance and simultaneously reduces magnetocrystalline anisotropy, leading to increased magnetic susceptibility at lower magnetic fields [68].

In this work, we employed a chemical engineering approach to tune the magnetic properties of spinel ferrite nanoparticles used as fillers in MAEs. Specifically, CoFe_2_O_4_, Zn_0.3_Co_0.7_Fe_2_O_4_, and Ni_0.3_Co_0.7_Fe_2_O_4_ nanoparticles were synthesized via the sol–gel auto-combustion method. This compositional tuning enabled control over the magnetic characteristics of the elastomer layer—particularly magnetic susceptibility and coercivity—which in turn allowed us to enhance the magnetoelectric response of a two-layer composite multiferroic system composed of a magnetic elastomer and a piezoelectric PVDF layer. The ability to tailor magnetoelectric coupling through nanoparticle composition opens up promising avenues for the development of flexible, lightweight devices for energy harvesting, magnetic field sensing, and self-powered electronics.

## 2. Materials and Methods

### 2.1. Synthesis of Magnetic Nanoparticles

For the synthesis of pristine and substituted cobalt ferrite magnetic nanoparticles, a sol–gel auto-combustion method was used [69]. To synthesize pristine cobalt ferrite (CoFe_2_O_4_, denoted as CFO), stoichiometric amounts of iron and cobalt nitrates (Fe: Co molar ratio = 2:1) were dissolved in 10 mL of distilled water. Concurrently, citric acid monohydrate, serving as both a chelating agent and fuel, was dissolved in a separate beaker containing 10 mL of distilled water. These solutions were admixed and subjected to continuous magnetic stirring at 150 °C for 2 h to promote metal–citrate complex formation. The pH of the solution was adjusted to ~7 with aqueous ammonia. Subsequently, the temperature was elevated to 300 °C to initiate the self-propagating combustion reaction, yielding a fine powder of CoFe_2_O_4_.

For the preparation of zinc-substituted cobalt ferrite (Zn_0.3_Co_0.7_Fe_2_O_4_, denoted as ZnCFO) and nickel-substituted cobalt ferrite (Ni_0.3_Co_0.7_Fe_2_O_4_, denoted as NiCFO), an identical synthetic procedure was followed, with appropriate stoichiometric adjustments to the metal precursors. Specifically, cobalt nitrate was partially replaced with zinc nitrate or nickel nitrate to achieve the target compositions while maintaining the total Fe:Me^2+^ ratio at 2:1 (where Me^2+^ represents the divalent metal ions Co^2+^, Zn^2+^, and Ni^2+^).

### 2.2. Synthesis of Magnetic Elastomers

The following materials were used for the synthesis of polymer matrix: α,ω-divinyl-polydimethylsiloxane (Vinyl silicone oil 5000 cSt, Penta-91, M_n_ = 37000), polymethylhydridosiloxane (P-804, Penta-91, M_n_ = 3600), α,ω-dihydrid-oligodimethylsiloxane (M_n_ = 1600), ω-vinyl-oligodimethylsiloxane (M_n_ = 2900), and 7% hexachloroplatinic acid solution in isopropyl alcohol (Speier’s catalyst) (all components from Sigma Aldrich, Saint Louis, MO, USA).

The synthesis of magnetoactive elastomers is described in detail in [70]. Briefly, four polymer components were first mixed together: 3 g α,ω-divinyl-polydimethylsiloxane (main polymer), 0.029 g polymethylhydridosiloxane (cross-linking agent), 0.097 g α,ω-dihydrid-oligodimethylsiloxane (chain length extender), and 1.01 g ω-vinyl-oligodimethylsiloxane (side chains). The prepared polymer mixture was poured into a Teflon Petri dish together with 16.6 wt.% of magnetic particles where it was mechanically mixed. Then, 30 μL of Speier’s catalyst solution was used per 1 g of magnetopolymer mixture. The Petri dish was then placed in an oven at a temperature of 90 °C for curing. After curing, the concentration of magnetic nanoparticles in MAE is 16 wt%. For the rheological measurements, disk-shaped samples with a diameter of 20 mm were cut. Samples of size 10 × 10 × 1 mm^3^ were cut for further assembly in a layered structure with PVDF.

**Figure 1:**
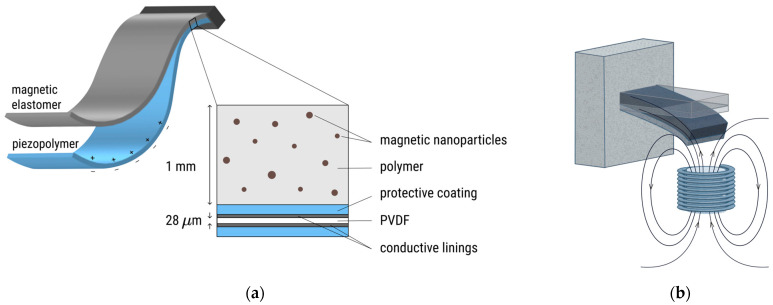
(**a**) A schematic representation of a two-layer PEP-MAE sample; (**b**) a schematic representation of a two-layer sample undergoing bending in a gradient magnetic field from a single electromagnet.

The structure employed for magnetoelectric effect (MEE) measurements consists of a piezopolymer layer PVDF (LTD0-028K, TE Instruments, Delft, The Netherlands) and a magnetic elastomer layer (see Figure 1a)—PEP-MAE structure. PEP-MAE is a two-layer structure where one layer is a PVDF piezopolymer with applied conductive linings and protective coating, and the second layer is a magnetic elastomer. In this case, the magnetic elastomer layer is a composite material layer based on a PDMS polymer and 16% magnetic nanoparticles. The layers are interconnected due to the lack of slippage.

### 2.3. Methods

Structural characterization of the synthesized magnetic nanoparticles was performed using X-ray diffraction (XRD) on an AXRD Benchtop powder diffractometer (Proto Mfg.,Taylor, MI, USA) with monochromatic Cu Kα radiation (λ = 1.540562 Å). The main diffraction maxima were indexed according to JCPDS card no. 22-1086.

The viscoelastic properties of MAEs were measured using an Physica MCR 302 rheometer (Anton Paar, Graz, Austria) with a plate–plate measuring system and an MRD 170/1 T magnetic cell equipped with an electromagnet. A disk-shaped MAE sample was placed between a stationary lower plate and an upper plate connected to a rotor. Shear harmonic oscillations were applied to the samples with angular frequency ω = 10 rad/s and strain amplitude of γ = 0.02% in the linear viscoelastic regime. The direction of the applied magnetic field is perpendicular to the plane of the MAE sample.

Measurements of magnetic properties were carried out with Vibration Sample Magnetometer (VSM) LakeShore 7407 (LakeShore Co., Westerville, OH, USA) at room temperature. Magnetic nanoparticles were placed inside Teflon capsula, which were attached to the holder of the VSM. The mass of one sample was near 1–3 mg. The sample of MAE with plane sizes 4 × 4 mm^2^ was attached to the holder; the mass of one sample was near 10–15 mg. The thickness of the MAE sample was 1 mm. The MAE sample was placed between electromagnet poles; the direction of magnetic field was parallel to the plane of the sample.

Measurements of magnetoelectric properties were carried out with a gradient AC magnetic field, with the setup described previously [62], and it was used without an external bias DC magnetic field. The cantilever configuration, namely, the configuration of the sample with one fixed end, was used as well. The frequency range was from 2 up to 200 Hz with 2 Hz increment. The amplitude of the AC magnetic field at the center of the free edge of the sample was calculated from the value of the current in the electromagnet using the calibration factor. The distance between the undeformed sample plane and electromagnet surface was 3 mm. A schematic representation of a two-layer sample undergoing bending in a gradient magnetic field from a single electromagnet is shown in Figure 1b.

## 3. Results

**Figure 2:**
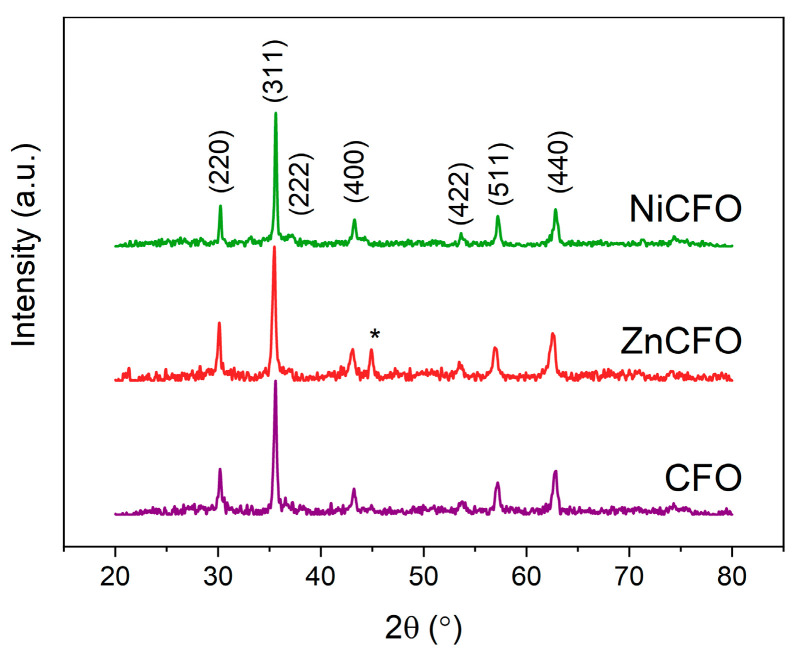
XRD patterns of CFO, ZnCFO, and NiCFO powders with indexed crystallographic planes corresponding to cubic spinel structure (space group $Fd\bar{3}m$). Asterisk (*) indicates (110) reflex corresponding to impurity phase.

The XRD patterns (Figure 2) confirmed the formation of a cubic spinel ferrite structure with the space group $Fd\bar{3}m$ as the predominant phase in all samples. For the ZnCFO sample, a minor reflection at 2θ ≈ 45° was observed, attributable to (110) reflection of a secondary phase (<8 at.%) with a body-centered cubic (bcc) structure, likely corresponding to metallic Fe or iron-rich FeCo alloy. Mean crystallite sizes were determined using the Scherrer equation applied to the five most intense diffraction peaks, yielding average values of 22 ± 3 nm for pristine CFO, 19 ± 5 nm for ZnCFO, and 26 ± 6 nm for NiCFO samples.

The lattice constant of the Zn-substituted sample (*a* = 8.394 Å) was slightly higher than that of the unmodified CFO (*a* = 8.365 Å), which is attributed to the substitution of Fe^3+^ ions (ionic radius ≈ 0.49 Å) with the larger Zn^2+^ ions (≈0.60 Å) in tetrahedral sites. This lattice expansion is a well known effect in Zn-substituted cobalt ferrites and is typically linked to changes in the inversion degree of the spinel structure [71]. Conversely, in the Ni-substituted sample, the lattice constant was slightly reduced (*a* = 8.357 Å), reflecting the smaller ionic radius of Ni^2+^ (≈0.69 Å) compared to Co^2+^ (≈0.75 Å), considering that both cations prefer octahedral sites.

**Figure 3:**
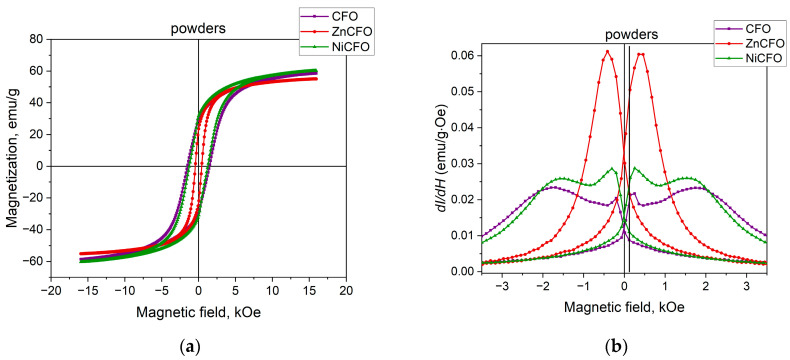
(**a**) Hysteresis loops and (**b**) first derivatives of hysteresis loops for powders of cobalt ferrite nanoparticles with partial substitution.

The magnetic properties of the three types of particles are presented in Figure 3. The saturation magnetization (*I*_s_) of the three samples is approximately the same. For the powder of ZnCFO, the coercive field of the hysteresis loop is the lowest, at about 400 Oe, whereas the coercive fields (*H*_c_) of the other powders exceed 1 kOe. The specific saturation magnetization, coercive field, and squareness ratio data (*I*_r_/*I*_s_) for the powders are summarized in Table 1. The squareness ratio reaches 0.5 for the two samples, the CFO and NiCFO powders, and it is smaller for the sample ZnCFO powder.

Figure 3b shows the first derivatives of the hysteresis loops (*dI/dH*), which qualitatively correspond to the magnetic susceptibility of the samples. The maximum magnetic field sensitivity for the CFO powder is observed at approximately 1.7 kOe, while partial substitution with Zn^2+^ or Ni^2+^ ions reduces this field to a range of 310–420 Oe. Moreover, for the NiCFO powder, the susceptibility remains near its maximum over a field range from 200 Oe to 2.5 kOe. Maximum values of the first derivative of the sample are presented in Table 1. Such an analysis of the magnetic characteristics allows for a more precise determination of the magnetic field ranges in which the magnetoelectric effect should be investigated. The nonmonotonic dependence of the susceptibility (observed for both CFO and NiCFO samples), featuring two peaks, may indicate the presence of two magnetic phases corresponding to different particle cluster sizes. The smaller particles, whose sizes match the crystallite dimensions, exhibit lower coercive fields, which is reflected in a peak in the susceptibility at low magnetic fields. Larger particle clusters contribute to higher coercive field values, resulting in a peak at higher magnetic fields.

The shear moduli were measured for MAE samples both in zero magnetic field and under an applied magnetic field of 1 T oriented perpendicular to the sample plane. The application of an external uniform magnetic field does not result in a dramatic change in the shear modulus of the samples. This indicates the absence of a magnetorheological effect in these elastomers containing nanosized particles. It is likely that the nanoparticles do not reorganize within the elastic matrix like microparticles. This lack of rearrangement is attributed to the small size of the particles—the mechanical moment resulting from magnetic dipole–dipole interactions is negligible compared to the restoring elastic forces of the polymer matrix. The shear modulus of the MAE samples with 16 wt% nanoparticles is 6 ± 1.2 kPa.

**Figure 4:**
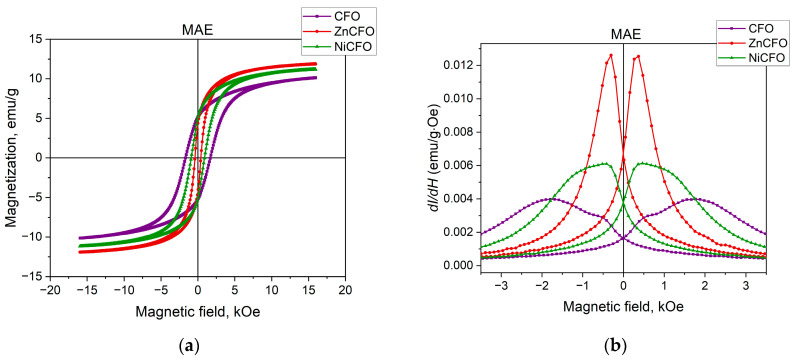
(**a**) Hysteresis loops and (**b**) first derivatives of hysteresis loops for magnetic elastomers containing CFO and partially substituted CFO nanoparticles.

Figure 4a,b presents the magnetic properties of the MAEs containing CFO and partially substituted CFO nanoparticles. All hysteresis loop parameters of the MAEs are provided in Table 1. The specific saturation magnetization of the magnetic elastomers is consistent with the approximately 16 wt% particle concentration, confirming the synthesis results. The sample of MAE with ZnCFO shows the smallest value of squareness ratio and coercivity. The coercive field of the ZnCFO-containing MAE sample almost coincides with that of the corresponding powder. The coercive fields for the MAEs containing CFO and NiCFO particles change relative to the powders. The remagnetization mechanism of coarse particle clusters in the elastic medium may involve particle rotation, which can lead to changes in the coercive field. The field dependence of the magnetic susceptibility of the MAEs also exhibits nonmonotonic behavior, although the maxima are more smoothed relative to the powders. This again suggests the presence of two phases with different particle size distributions. The maximum susceptibility is observed for the elastomer with ZnCFO particles; all values are presented in Table 1.

**Figure 5:**
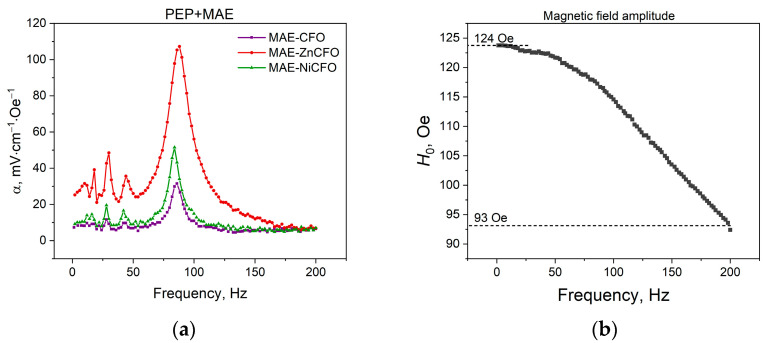
(**a**) Frequency dependence of magnetoelectric coefficient for layered structure with magnetic elastomers containing partially substituted cobalt ferrite nanoparticles (measured in gradient alternating magnetic field), and (**b**) frequency dependence of magnetic field amplitude produced by electromagnet at distance of 3 mm from its surface.

Figure 5a presents the frequency dependence of the magnetoelectric (ME) coefficient for the two-layer PEP–MAE samples. The ME coefficient α was calculated using the following standard formula:

(1) $$\alpha =\frac{U}{t\cdot H}\cdot [\mathrm{mV}\cdot {\mathrm{cm}}^{-1}\cdot Oe^{-1}],$$

where U is the induced voltage, *t* is the thickness of the piezoelectric polymer, and *H* is the average magnetic field, determined at a distance of 3 mm from the electromagnet surface. Figure 5b shows the frequency dependence of the magnetic field amplitude. In calculating *α*(*f*), each frequency value *f* corresponds to a specific magnetic field *H*, as determined from this dependence.

The magnetic elastomer layer is drawn into the field gradient, which deforms the piezoelectric layer and induces an electrical voltage. A two-layer sample with ZnCFO nanoparticles in the magnetoelastomer layer, exhibiting the highest magnetic susceptibility and lowest coercive field, shows the highest values of the magnetoelectric effect—up to 107 mV·cm^−1^·Oe^−1^. Conversely, the sample with unmodified CFO particles exhibits lower susceptibility, higher coercive fields, and correspondingly lower MEE values. The sample with NiCFO nanoparticles (PEP-MAE-NiCFO) shows an increase in the MEE compared to the sample PEP-MAE-CFO and a decrease in the MEE compared to the sample PEP-MAE-ZnCFO. This is due to the fact that the coercivity of MAE-NiCFO is smaller than for MAE-CFO and its susceptibility is larger. At the same time, *H*_c_ (MAE-NiCFO) > *H*_c_ (MAE-ZnCFO) at lower susceptibility. The resonant frequency (*f*_rez_) for all three samples coincides within experimental error (values are given in Table 2), which is attributed to their similar geometric parameters and nearly identical elastic moduli.

Previously, a mechanism for the magnetoelectric effect in PEP–MAE structures was proposed [62]. This mechanism is related to the action of the magnetic field gradient on the particles that fill the magnetic elastomer (f~m·∇H, where *f*—force, *m*—magnetic moment, *H*—magnetic field). The particles are drawn into the field gradient, causing the MAE layer to bend and, correspondingly, the PEP layer to deform, which induces an electrical voltage in the piezoelectric polymer. In earlier studies, elastomers containing iron microparticles were investigated [60,62]. In those systems, magnetodeformation and magnetorheological effects were observed in a homogeneous magnetic field—the restructuring of particles due to induced dipole–dipole interactions results in the elongation of the elastomer and changes in its elastic modulus [70]. However, in the present work, it is suggested that the restructuring of nanoparticles in the elastic polymer is negligible under an external magnetic field, and no magnetorheological effect is observed. Nevertheless, the bending of the elastomer in a gradient magnetic field still occurs, meaning that the drawing-in of particles remains significant even in the absence of their displacement.

The results obtained expand the range of control over the magnetoelectric effect by employing various filler particles in the MAE. When comparing these findings with previously published data [60,62] for structures with iron microparticles, it can be noted that the magnitude of the MEE is influenced by the magnetic properties, thickness, and elastic modulus of the magnetoelastomer layer. In the case of microparticles, their restructuring in the polymer matrix can affect the magnitude of the effect; however, with nanoparticles, such restructuring may not occur, yet the magnetoelectric effect still arises. Partial substitution of Co^2+^ ions with Zn^2+^ or Ni^2+^ ions modifies the magnetic properties, leading to an enhanced magnetoelectric effect at fixed elastic parameters. The result obtained from this study demonstrates the viability of employing nanoparticles for MEE in polymer layered structures of the proposed type.

For comparison, in [62], the maximum effect of 0.77 V∙cm^−1^∙Oe^−1^ was obtained in a sample based on PVDF and MAE with 77 wt% iron microparticles; the thickness of MAE layer was 3 mm, as well as 0.11 V∙cm^−1^∙Oe^−1^ in the same structure with the thickness of the MAE layer as 0.3 mm. The order of magnitude of the MEE obtained in this work is in agreement with the previous results, but the nanoparticle concentrations are significantly lower. In inhomogeneous magnetic fields without using a bias field, an MEE of 0.24 V∙cm^−1^∙Oe^−1^ was obtained in a b-LN/Ni/Metglass composite structure [11]; however, no resonance enhancement is observed in the frequency range of 10–1000 Hz in this measurement configuration. Similar order of magnitude MEEs of 30–750 mV∙cm^−1^∙Oe^−1^ were observed in a PVDF thin film with CFO nanoparticles depending on the nanoparticle concentration in homogeneous magnetic fields [51]. The MEE in the BaFe_0.01_Ti_0.99_O_3_ composite [72] was also investigated in uniform fields, which was 50–60 mV∙cm^−1^∙Oe^−1^. It should be noted that a variety of mechanisms of magnetoelectric conversion are present in each distinct case. These mechanisms are initiated under differing external conditions, thus prohibiting the comparison of results by absolute value. In the present work, magnetoelectric conversion in a non-homogeneous magnetic field is obtained, thus extending the potential of their application in the domains of energy harvesting and sensor technologies. Specifically, the alterations in the resonance frequency and magnitude of MEE can serve as indicators of changes in the inhomogeneity of the field, and consequently, alterations in the system as a whole. The potential for energy accumulation and its subsequent conversion into an electrical signal suggests the feasibility of a multifunctional device utilizing composite magnetoelectric principles for energy harvesting applications.

## 4. Conclusions

A study of polymer composites consisting of two layers—piezopolymer and magnetic elastomer—based on cobalt ferrite (CoFe_2_O_4_) nanoparticles and Zn- or Ni-substituted CoFe_2_O_4_ was carried out. Despite the absence of the observed magnetorheological effect and the supposed insignificant reorganization of nanoparticles under the influence of a DC external magnetic field, the magnetoelectric effect in a gradient AC magnetic field is confirmed. The highest magnetoelectric effect was found for the composite with ZnCFO up to 107 mV·cm^−1^·Oe^−1^, with its magnetic parameters exhibiting the lowest coercivity and the highest susceptibility. Thus, the substitution of Co^2+^ ions with Zn^2+^ or Ni^2+^ ions can improve the magnetic properties and increase the magnetoelectric effect at fixed elastic characteristics. The obtained results open new directions for controlling magnetoelectric effects in such composite materials, expanding their potential applications in energy harvesting and sensor technologies.

## Figures and Tables

**Table 1:** Parameters of hysteresis loop of powders and MAEs based on them.

Sample	*I*_s_, emu/g	*H*_c_, Oe	*I*_r_/*I*_s_	*dI*/*dH* (Max), emu/g∙Oe
Powder CFO	58.6 ± 0.3	1498 ± 10	0.51	0.023
Powder ZnCFO	55 ± 0.3	433 ± 2	0.42	0.061
Powder NiCFO	60 ± 0.3	1250 ± 8	0.52	0.028
MAE-CFO-16	10.1 ± 0.1	1683 ± 10	0.51	0.004
MAE-ZnCFO-16	11.9 ± 0.1	401 ± 2	0.36	0.013
MAE-NiCFO-16	11.2 ± 0.1	899 ± 6	0.46	0.006

**Table 2:** Parameters of dynamic magnetoelectric effect for PEP-MAE samples.

Sample	α, mV∙cm^−1^∙Oe^−1^	*f_rez_*, Hz
PEP-MAE-CFO	31.5 ± 0.3	86 ± 2
PEP-MAE-ZnCFO	107.3 ± 1.1	88 ± 2
PEP-MAE-NiCFO	84.0 ± 0.8	84 ± 2

## Data Availability

Data are contained within the article.
